# The Effect of Grit on Career Adaptability of Chinese College Students Based on the Self-Regulatory Processes

**DOI:** 10.3389/fpsyg.2021.795153

**Published:** 2021-12-08

**Authors:** Haihong Li, Xuan Yu, Yuanfei Mei, Xuhong Liu, Ling Li, Nan Luo

**Affiliations:** ^1^School of Business Administration, Chongqing Technology and Business University, Chongqing, China; ^2^School of Life Science and Technology, University of Electronic Science and Technology, Chengdu, China; ^3^School of Management Science and Engineering, Chongqing Technology and Business University, Chongqing, China; ^4^Department of Police Management, Sichuan Police College, Luzhou, China

**Keywords:** grit, career adaptability, self-regulatory process, positive affect, goal commitment

## Abstract

Intelligence is innate, but grit is something everyone can develop. Grit not only enables students to stick to their goals, but also to persevere even when they fail. Career adaptability is an important concept in vocational education of college students, which is a person engaged in some work, must have a certain physical and psychological quality. Base on the self-regulation theory, this study investigated the relationship between grit and career adaptability of Chinese college student based on the self-regulatory processes. We surveyed 839 Chinese college students and tested a self-regulation model. As expected, grit was related to greater career adaptability *via* greater career exploration and decision self-efficacy, positive affect, and goal commitment. These findings not only broaden the theoretical framework for the effect of grit on career adaptability, but also open up a new horizon for improving college students’ career adaptability in practice.

## Introduction

The study of character strengths, such as grit and resilience, takes center stage in human resource management and occupational psychology, not only to improve productivity, but also to promote wellbeing and engagement in the workplace (Niemiec, 2017; Ting and Datu, 2020). Since grit is a relatively fresh concept, it has been defined and studied in the last recent years, and so far there has been growing interest and enthusiasm in cultivating personal grit (Tough, 2012; Kim and Kong, 2021), it is essential to continue studying grit in different contexts. Grit is defined as a quality of persisting in working hard and never giving up in the face of goals (Duckworth et al., 2007). Studies have examined that grit is able to have positive impact on several fields related to achievement: college grades, high school graduation, and so on (Eskreis-Winkler et al., 2014). What is more, some studies introduce grit into the field of career development (Suh, 2020; Olckers and Koekemoer, 2021). It is important to study the mediating mechanism between grit and career adaptability, because career development is becoming more irregular and boundaryless due to corporate change, economic and social insecurity, and changes in employment patterns (Guan et al., 2013; Kundi et al., 2021; Lee and Moon, 2021).

College students are more likely to experience job insecurity during the transition from student to workplace, which can affect personal and mental health (Wibowo et al., 2021). For people in marginal social status, such as college students, they often feel that their future career is fraught with risk (Seiffge-Krenke et al., 2012). What is more, compared with the knowledge content of the student stage, in an increasingly unstable work environment, students’ career adaptability is indispensable (Guan et al., 2013). Therefore, many scholars believe that students need to cultivate positive psychological ability to respond to the changing career tasks and environment (Nilforooshan and Salimi, 2016; Jeong, 2019).

Previous studies have studied the relationship between grit and career adaptability based on career construction theory, most of which are based on the samples of college students from western countries (Datu et al., 2018a; Gregor et al., 2021). They both believed grit is very important to the career development among college students. Because grit requires demonstrating passionate perseverance and adaptability with long-term goals, it can serve as a means to achieve important career development milestones and positively predict career adaptability (Park and Yang, 2020; Gregor et al., 2021). So far, there is not much research has explored the mediating mechanism of the relationship between grit and career adaptability, previous studies have shown that grit influences career adaptability through resilience and creativity (Han, 2018; Jeong, 2019). In order to broaden the theoretical framework of the influence of grit on career adaptability, and the applicability of the relationship between college students’ grit and career adaptability in China, we investigated the mediating mechanism between grit and career adaptability from the perspective of self-regulatory process (SRP).

Career adaptation is a dynamic and SRP (Savickas, 1997). Young people will do their best to regulate their cognition, affect, and motivation during the transition from school to the workplace (Wibowo et al., 2021). Specifically, when faced with unsatisfactory progress or career problems, young people may re-evaluate their abilities to achieve future career goals (i.e., career exploration and decision self-efficacy), manage the emotions associated (i.e., emotions), and lose momentum in pursuing present goals (i.e., goal commitment). For example, during a job search, a college graduate failed to join the company he wanted, if he has a strong self-regulatory ability, he will re-evaluate their abilities, rational thinking and maintain the positive emotions, find the problem from the failure, will also strengthen their present goals, and keep on trying until he finds another satisfactory job. In fact, this phenomenon is also concerned and studied by scholars. Most scholars emphasize that the SRP focuses on self-observation, self-judgment, and the management of related emotions (Diefendorff and Lord, 2008; Zacher, 2014). Studies have shown that grit is an important factor that governs and regulates human behavior (Hu et al., 2017; Min, 2018). In addition, many studies have demonstrated that the SRP can positively predict career adaptability (Schraub et al., 2011; Jundt et al., 2014). This suggests that SRPs involving cognition, affect, and motivation may play a role together between grit and career adaptability. Therefore, we hypothesize that college students with high grit will allocate cognitive, emotional, and motivational resources to deal with all kinds of situations in their future career environment. However, students with low grit may lose confidence and no longer stick to their goals when they encounter difficulties and setbacks. To test the hypothesis, we introduce career exploration and decision self-efficacy(cognition), positive affect(emotional), and goal commitment(motivational) as three parallel mediators to study the relationship between grit and career adaptability.

This study contributes to the existing literature in the following two aspects. First, by analyzing the mechanism of Chinese college students’ grit on career adaptability, this study expands the application of self-regulation process in this field and opens the mediating the mechanism of college students’ grit on career adaptability, which is beneficial to college students to adopt self-regulation behaviors when facing career changes or unpredictable career problems to better cope with the challenges in the future workplace. On the other hand, we examine the relationship between grit and career adaptability of Chinese college students, which broadens the application prospect of the theoretical model. In conclusion, we inspire colleges to particularly emphasis the cultivation of grit in career guidance education through the study grit and career adaptability. It also guides students to give full consideration to their own abilities and social environment when making career planning, show passion and persistence to long-term goals, face life and work with a positive attitude, and help students to better transition to work in the future.

## Literature Review and Hypothesis

### Grit and Career Adaptability

Grit means the passion and perseverance shown to achieve a long-term goal, it is characterized by long-term efforts to achieve goals, trying to conquer challenges and deal with difficulties, working hard, and finally being able to achieve one’s desired goals. On the other hand, consistency of interest is characterized by having a clear goal, not being easily shift and changing goals, and having a lasting desire (Duckworth et al., 2007). Previous research has shown that grit has positive effects to meaning in life (Datu et al., 2018b), career motivation (Park and Lee, 2020), and career adaptability (Meriac et al., 2015). However, it remains to be studied whether grit can positively predict career adaptability in the Chinese context. Therefore, we introduce the theoretical model of grit and career adaptability into China to predict whether Chinese college students with grit can better cope with adverse and stressful situations and adapt to changing environments more easily. Here, career adaptability is defined as the core competencies that an individual to respond various challenges brought about by job or role changes when unforeseen events change their career plans (Savickas, 1997).

College students who have more grit are shown to have clear ideas, always participating in activities that support their goals (Lee and Sohn, 2017). They will have a unique set of strategies for coping with career challenges, allowing them to face difficult situations in a less stress way and not give up easily goals (Yoon et al., 2017; Jang and Huh, 2019). Students with high grit will start to plan their goals from young age, beginning of choosing majors that suit their own wants and interests, and they will try to get high marks and achieve their goals (Jiang et al., 2019). Therefore. cultivating grit can be used as a way for the Chinese college students to better adapt to the transition from school to career (Koen et al., 2012; Datu et al., 2016). According to the career construction theory and the career adaptability model developed on the basis of it, the process of college students’ career adaptation is to acquire the characteristics of adaptation, take corresponding behaviors, and improve the adaptability to achieve the result of adaptation (Savickas, 2002). Consequently, we propose the hypotheses 1:

*Hypothesis 1*: Grit will have a significant positive effect on Chinese college students’ career adaptability.

### The Effect of Grit on Career Adaptability Based on the Self-Regulatory Processes

The process of college students adapting to different activities and behaviors in their career is a process of self-regulation (Bandura, 1991). The self-regulation theory (Bandura, 1991) points out that the SRP is to set and gradually achieve individual goals and generate thoughts, emotions, and behaviors. A college student’s ability to regulate himself or herself is determined by his or her sustained enthusiasm for his or her goals and by the amount of effort he or she puts into coping with changing environment (Wolters and Hussain, 2015). This is in line with the personality traits of the gritty (Duckworth et al., 2007). In other words, we think that college students with grit are willing to put in the effort and passion to pursue their careers by regulating their emotions, cognition, and motivation. Previous studies have shown that individuals who lack of grit is related to inadequate self-regulation (Gupta and Sudhesh, 2019). In order to further study, the mediating mechanism of grit on career adaptability, we conducted research specifically from the cognition (career exploration and decision self-efficacy), emotion (positive affect), and motivation (goal commitment) of Chinese college students.

In the career development theory, it is clearly pointed out that individual factors (such as individual personality characteristics) affect the development of career exploration and decision self-efficacy, but it does not specify the details of these factors (Jordaan, 2008). Kundu (2017) believed that students with high grit will have higher personal agency to challenge expected failures and have the confidence to explore various tasks in their future careers, and overcome obstacles to academic and career success (Kundu, 2017). In addition, a study points out that personality traits are a significant individual factor affecting career exploration (Fan et al., 2012; Ireland and Lent, 2018). Since grit is a positive personality traits that individuals exhibit in the process of growth and development (Duckworth et al., 2007), studies have shown that grit have positive impact on self-efficacy (Lim et al., 2016; Joy et al., 2020; Shin and Joo Ram, 2020). In order to improve the model structure, we will take self-efficacy into career field to study the effect of grit on career exploration and decision self-efficacy. Therefore, we assume that as:

*Hypothesis 2*: Grit will be positively associated with career exploration and decision self-efficacy.

College students are at a critical stage of life. Positive affect can make people think that the future is full of opportunities and hopes, so they tend to take the initiative (Maddi et al., 2013). Positive affect is unique responses to things that are personally meaningful (Fredrickson, 1998). Positive affect can expand the thinking and action range of individuals, and then construct durable personal resources (intellectual resources, physical resources, psychological resources, and social resources), thus bringing long-term adaptive advantage (Fredrickson, 2001). According to the broaden-and-build theory of positive emotions, the direction of emotional response is influenced by personal traits (Luthans et al., 2007). As an excellent trait of college students, grit may induce their emotional experience of positive affect. Studies have proved that people with high grit are better able to cope with stress and maintain mental health (Salles et al., 2013; Meriac et al., 2015), and grit is positively correlated with wellbeing (Lucas et al., 2015). We think that the more grit college students are, the more positive affect they experience. First of all, college students with high grit have strong self-determination consciousness and high psychological quality, which promote positive changes in their emotional level, produce more pleasure perception, and bring more positive emotional experience (Lavy and Littman-Ovadia, 2016). What is more, grit can promote the establishment and accumulation of psychological resources of college students. The more psychological resources an individual has, the less stress he or she faces in people’s life, work and study and can use these resources to prevent pressure from turning into negative affect, such as anxiety, so as to show more positive affect (Meyers and van Woerkom, 2017). Therefore, we suppose as:

*Hypothesis 3*: Grit has a significant positive effect on positive affect.

Hollenbeck and Klein (1987) defined goal commitment as an individual’s strong willingness to take actions within a certain period of time and move closer to the goal so as to achieve the goal (Hollenbeck and Klein, 1987). The gritty has sustained passion for goals and lasting endurance (Alhadabi and Karpinski, 2019). The research of Duckworth et al. (2007) found that the outstanding people in finance, art, academic, and other industries all have the personality trait of grit. It is because of this trait that individuals can face difficulties and setbacks and bottlenecks head-on, still maintain lasting enthusiasm for the goal, and make unremitting efforts to finally reach their goals (Eskreis-Winkler et al., 2014; Cho, 2020).

In the process of achieving career goals, college students will meet with all kinds of difficulties, the individual may inadvertently will reduce the standard of the goal. If college students have lasting passion and perseverance for their career goals, they may have a high commitment to the set career goals when performing their duties, and will take the initiative to overcome the obstacles to reach the original goals by extending their working hours and enhancing their working skills. Previous studies have found that compared with individuals with low grit, individuals with high grit can achieve better academic achievement (Cross, 2014; Hodge et al., 2018; Tang et al., 2021) and persisted longer on tasks that were at risk of failure (Guerrero et al., 2016). Therefore, we believe that the more gritty college students are, the more willing they are to stick to a certain career goal and will not change them easily. We propose the following hypothesis:

*Hypothesis 4*: Grit has a significant positive effect on goal commitment.

Firstly, college is a period of career exploration for young people and the starting point of their future life (Osipow and Fitzgerald, 1996). In this stage, college students explore suitable career activities through the recognition and understanding of their role and environment (Schraub et al., 2011). Previous studies have shown that negative emotions, such as decision anxiety, have a negative impact on career adaptability and career decision self-efficacy can significantly predict the development of career adaptability of college students (Anderson and Mounts, 2012). The more confident individuals are in their decision ability, the more likely they are to seek employment information, the more likely they are to maintain a high level of persistence to resist external pressure, and the more involved they are in career planning and exploration, which corresponds to the definition of career adaptability (Hu et al., 2016). In other words, career exploration and decision self-efficacy can positively affect career adaptability.

Secondly, as an individual’s ability to maintain balance in changes, career adaptability can help college students actively adapt to changing environment and role requirements in different stages of career development (Savickas, 2002). In this process, college students need to have a clear understanding of themselves and career goals in a new environment, and have a positive mood for their future career development. The first reason is that having positive affect can make individuals more creative and better at solving problems. In addition, positive affect can help individuals construct lasting psychological resources and have more ways to carry out career exploration and development (Coetzee and Harry, 2014). The research proved that both emotional stability and emotional intelligence can positively influence career adaptability (Huang et al., 2014). Therefore, in this study, we also believe that positive affect can significantly influence career adaptability.

Thirdly, there will be a series of career goals in the process of career development, and achieving career success usually requires the continuous realization of these goals. Furthermore, Lent et al. (1994) believed that goals are important parts of a career, with career planning, ambition, and choices embedded in basic goal mechanisms. In the career construction theory, Savickas (2002) further recognized the significance of career different goals in the behavioral self-regulatory of individuals in coping with various work tasks and role changes. They argue that by setting and committing various career goals, individuals can better regulate and guide their behavior motivating themselves, and increasing their ability to cope in the face of career changes or unpredictable career problems (Kim and Kim, 2018). As an important attitude in goal management, goal commitment has an important influence on career development (Burkley et al., 2013). So goal commitment positively affects career adaptability.

How does the grit influence career adaptability of Chinese college students? These problems have not been fully studied at present. Self-regulation theory explains how people direct their motivations, thoughts, and actions in pursuit of happiness, comfort, and adaptation to their environment (Sandars and Cleary, 2011). It has been pointed out that the SRP is considered to be an important part of studying the relationship between different personality traits and professional behavior (Liveris and Cavanagh, 2012; Nilforooshan and Salimi, 2016). Many studies suggest that SRPs play mediating roles (Gellert et al., 2012; Praskova et al., 2014; Hu et al., 2018). For example, Hu et al. (2018) believed that SRPs mediate the effect of negative career feedback on career goal shifting and career exploration (Hu et al., 2018). Therefore, in order to further understand the mediating mechanism between grit and career adaptability, we combined the self-regulation theory with the above discussion to explore whether the SRPs (career exploration and decision self-efficacy, positive affect, and goal commitment) can mediate the relationship between grit and career adaptability through the study of Chinese college students as sample. Therefore, we assume that as:

*Hypothesis 5*: Career exploration and decision self-efficacy act as a mediator variable between grit and career adaptability.

*Hypothesis 6*: Positive affect mediates the relationship between grit and career adaptability.

*Hypothesis 7*: Goal commitment plays a mediating effect on the relationship between grit and career adaptability.

## Materials and Methods

### Participants and Procedures

A cluster random sampling method was used to select 1,000 students from a college in Chongqing, China. In order to prevent the deviation of homologous methods, we conducted a longitudinal study design. Besides, this research has set up one item separately: the last four digits of the mobile phone number, so that the data corresponding to the above variables can be effectively matched.

SPSS 21.0 and AMOS 17.0 are used to analyze and process the data. The hierarchical regression method was used to analyze the main effect and the moderating effect, Hayes’ Process macro plug-in was used to test the mediating effect (Hayes, 2015). At the same time, structural equation model was used to test the scale structure validity and path coefficient.

A total of 1,000 complete questionnaires were obtained by matching the last four digits of the mobile phone number, after incomplete questionnaires had been excluded, there were 893 (89.30%) valid responses. The characteristics of the sample data are shown in Table 1.

**Table 1:** Demographic information of participants.

		*N*	Proportion (%)
Gender	Male	206	23.1
	Female	683	76.9
Age	Under 18	95	10.6
	Between 19 and 20	506	56.7
	21 and above	292	32.7
Nationality	Han nationality	803	89.9
	Other ethnic minorities	90	10.1
Grade	Freshmen	400	44.8
	Sophomores	203	22.7
	Juniors	266	29.8
	Seniors	24	2.7
Parents’ education	Junior high school or below	541	60.6
	Senior high school, technical secondary school or technical school	222	24.9
	Junior college	67	7.5
	Undergraduate	59	6.6
	Postgraduate	4	0.4
GPA of last academic year	less than or equal to 2.0	65	7.3
	Greater than 2.0 and less than or equal to 2.5	117	13.1
	Greater than 2.5 and less than or equal to 3.0	180	20.2
	Greater than 3.0 and less than or equal to 3.5	273	30.6
	Greater than 3.5 and less than or equal to 4.0	189	21.2
	Greater than 4.0 and less than or equal to 4.5	55	6.2
	Greater than 4.5 and less than or equal to 5.0	14	1.6
Subjective social status	1	11	1.2
	2	33	3.7
	3	106	11.9
	4	159	17.8
	5	281	31.5
	6	170	19.0
	7	72	8.1
	8	43	4.8
	9	7	0.8
	10	11	1.2

In measuring subjective social status, 1 is the lowest, 10 is the highest, and the higher the score, the higher the status.

### Measures

In this study, we adopted the mature western scales to measure the variables. For ensuring the consistency and applicability of the English scale in the Chinese context, the author conducted a translation-back translation procedure (Brislin, 1986). Before the formal investigation, a preliminary test was conducted on 15 college students, and the items were modified according to their feedback.

#### Grit

Grit was measured with a 8-items scale developed by Duckworth and Quinn (2009). Responses were on a five-point Likert scale ranging from 1 (not like me at all) to 5 (very much like me), which includes two dimensions: consistency of interest (e.g., “I often set a goal but later choose to pursue a different one”) and perseverance of effort (e.g., “I finish whatever I begin”). Drawing on previous studies (e.g., Wibowo et al., 2021), we did not distinguish the two dimensions of grit in our study, and took them as a whole indicator. Cronbach’s alpha was 0.60.

#### Career Exploration and Decision Self-Efficacy

Career exploration and decision self-efficacy were measured with a 12-items scale developed by Lent et al. (2015). Responses were asked to answer “How much confidence do you have in your ability to …” on a 10-point Likert scale ranging from 1 (no confidence at all) to 10 (full confidence), which includes two dimensions: decisional self-efficacy (e.g., “identify careers that best use your skills”) and coping efficacy (e.g., “cope with the disappointment if your first choice does not work out”). Drawing on previous studies (e.g., Wolf et al., 2021), we did not distinguish the two dimensions of career exploration and decision self-efficacy in our study, and took them as a whole indicator. Cronbach’s alpha was 0.96.

#### Positive Affect

Positive affect was measured with the 6-items tense-calm scale dimension in affective wellbeing scale developed by Warr (1990). Responses were asked to answer “Thinking of the past few weeks, how much of the time has your job made you feel each of the following?” Responses were as: never, occasionally, some of the time, much of the time, most of the time, and all of the time; and answers were scored from 1 to 6, respectively. The item was as: Tense, Uneasy, Worried, Calm, Contented, and Relaxed. Cronbach’s alpha was 0.67.

#### Goal Commitment

Goal commitment was measured with the 9-items scale developed by Hollenbeck et al. (1989). Responses were on a five-point Likert scale ranging from 1 (not like me at all) to 5 (very much like me). The sample item was “I am strongly committed to pursuing this GPA goal.” Cronbach’s alpha was 0.74.

#### Career Adaptability

Career adaptability was measured with the 24-items scale developed by Hou et al. (2012). Responses were on a five-point Likert scale ranging from 1 (not strong) to 5 (strongest), which includes four dimensions: Concern (e.g., “Thinking about what my future will be like”), Control (e.g., “Keeping upbeat”), Curiosity (e.g., “Exploring my surroundings”), and Confidence (e.g., “Performing tasks efficiently”). Drawing on previous studies (Guan et al., 2013), we did not distinguish the four dimensions of career adaptability in our study, and took them as a whole indicator. Cronbach’s alpha was 0.97.

#### Control Variables

The control variables were selected based on previous studies related to career adaptability. These include gender, age, grade, nationality, GPA of last academic year, subjective social status, and parents’ education.

Based on previous studies, firstly, we collected the general demographic information about the participants, including gender, age, grade, nationality, GPA of last academic year (Goodale and Hall, 1976; Duckworth et al., 2009; Zacher, 2014; Denault et al., 2018) to control for their effects on students’ grit, and career adaptability. Second, as students’ subjective social status can also influence their career adaptability behavior (Autin et al., 2016). we included students’ subjective social status as a control variable when predicting grit and youths career adaptability. Lastly, according to the research of Guan et al. (2018), we also take the parents’ education level as the control variables (Guan et al., 2018).

## Result

### Descriptive Statistics

Table 2 presents the descriptive statistics and correlations for the study variables. Grit correlated moderately with career exploration and decision self-efficacy (*r* = 0.211, *p* < 0.01), and slightly with positive affect (*r* = 0.092, *p* < 0.01) and goal commitment (*r* = 0.087, *p* < 0.01), and moderately with career adaptability (*r* = 0.296, *p* < 0.01). Career exploration and decision self-efficacy, positive affect, and goal commitment correlated with career adaptability (*r* = 0.430, *p* < 0.01; *r* = 0.112, *p* < 0.01; *r* = 0.286, *p* < 0.01).

**Table 2:** Descriptive statistics and correlations of study variables.

S. No	Variables	*M*	*SD*	1	2	3	4
1.	Grit	3.294	0.409				
2.	Career exploration and decision self-efficacy	6.442	1.223	0.211^**^			
3.	Positive affect	3.515	0.220	0.092^**^	0.017		
4.	Goal commitment	3.395	0.486	0.087^**^	0.216^**^	0.041	
5.	Career adaptability	3.850	0.589	0.296^**^	0.430^**^	0.112^**^	0.286^**^
6.	Gender	0.770	0.422	−0.042	−0.023	−0.019	0.098^**^
7.	Age	2.230	0.645	−0.022	−0.010	0.053	0.001
8.	Grade	1.900	0.919	−0.004	0.057	0.048	0.025
9.	Nationality	1.110	0.369	0.003	−0.045	−0.030	0.005
10.	GPA of last academic year	3.700	1.375	0.030	0.037	0.045	0.122^**^
11.	Subjective social status	5.010	1.602	0.041	0.215^**^	0.000	0.114^**^
12.	Parents’ education	1.610	0.915	0.033	0.054	0.010	−0.002

*N* = 893,

** *p* < 0.01.

### The Predictive Effect of Grit on Career Adaptability

After controlling gender, age, grade, nationality, GPA of last academic year, subjective social status, and parents’ education, a hierarchical regression model was established with career adaptability as the dependent variable and grit as the independent variable. The results showed that grit significantly affected career adaptability (*β* = 0.417, *p* < 0.001), Hypothesis 1 is verified.

According to the hypothesis of this study, after controlling control variables, independent variables and dependent variables are gradually added, and the results are shown in Table 3. Among them, grit significantly affected career exploration and decision self-efficacy (*β* = 0.596, *p* < 0.001), Hypothesis 2 is verified. Grit significantly affected positive affect (*β* = 0.049, *p* < 0.01), Hypothesis 3 is not verified. Grit significantly affected goal commitment (*β* = 0.099, *p* < 0.05), Hypothesis 4 is verified. Career exploration and decision self-efficacy significantly affected career adaptability (*β* = 0.200, *p* < 0.001). Positive affect significantly affected career adaptability (*β* = 0.297, *p* < 0.001). Goal commitment significantly affected career adaptability (*β* = 0.337, *p* < 0.001). After the addition of career exploration and decision self-efficacy, the influence of grit on career adaptability decreased significantly (*β* = 0.311, *p* < 0.001), and the influence of career exploration and decision self-efficacy on career adaptability still had a significant positive effect (*β* = 0.178, *p* < 0.001), Hypothesis 5 is verified. After the addition of positive affect, the influence of grit on career adaptability decreased significantly (*β* = 0.406, *p* < 0.001), and the influence of positive affect on career adaptability still had a significant positive effect (*β* = 0.228, *p* < 0.001), Hypothesis 6 is verified. After the addition of goal commitment, the effect of grit on career adaptability decreased significantly (*β* = 0.386, *p* < 0.001), and goal commitment still had a significant positive effect on career adaptability (*β* = 0.309, *p* < 0.001), Hypothesis 7 is verified.

**Table 3:** Results of hierarchical regression analysis.

	Career exploration and decision self-efficacy	Positive affect	Goal commitment	Career adaptability						
	M1	M2	M3	M4	M5	M6	M7	M8	M9	M10
Control variable										
Gender	0.001	−0.009	0.125	−0.008	−0.019	−0.021	−0.064	−0.008	−0.005	−0.046
Age	−0.148	0.013	−0.023	−0.053	−0.030	−0.066	−0.054	−0.027	−0.056	−0.046
Grade	0.144^*^	0.004	0.016	0.052	0.025	0.054	0.049	0.026	0.051	0.047
Nationality	−0.132	−0.02	0.017	−0.008	0.019	−0.002	−0.013	0.015	−0.004	−0.014
GPA of last academic year	0.015	0.006	0.04	0.000	0.000	0.002	−0.01	−0.002	−0.001	−0.012
Subjective social status	0.154^***^	−0.001	0.035	0.051^***^	0.023^*^	0.055^***^	0.043^***^	0.024	0.052	0.041
Parents’ education	0.049	0.002	−0.007	−0.008	−0.014	−0.003	−0.001	−0.016	−0.008	−0.006
Independent variable										
Grit	0.596^***^	0.049^**^	0.099^*^	0.417^***^				0.311^***^	0.406^***^	0.386^***^
Mediating variable										
Career exploration and decision self-efficacy					0.200^***^			0.178^***^		
Positive affect						0.297^***^			0.228^**^	
Goal commitment							0.337^***^			0.309^***^
*R* ^2^	0.097	0.014	0.046	0.111	0.190	0.040	0.102	0.235	0.118	0.173
△*R*^2^	0.040	0.008	0.007	0.083	0.163	0.012	0.074	0.124	0.109	0.062
*F*	11.822^***^	1.592	5.271^***^	13.814^***^	25.986^***^	4.598^***^	12.549^***^	30.106^***^	13.164^***^	20.540^***^

*N* = 893,

* *p* < 0.05,

** *p* < 0.01,

*** *p* < 0.001.

According to the suggestion of Hayes (2015), the 95% confidence interval of the mediating effect was further calculated using the percentile Bootstrap method with bias correction, and 5,000 Bootstrap samples were selected from the samples (*n* = 839) to test the mediating effect. The results of total effect, direct effect, and indirect effect under the mediating effect are shown in Table 4. The total indirect effect value was 0.130, and the 95% confidence interval of the mediating effect path of career exploration and decision self-efficacy was 0.052 and 0.141, excluding 0, indicating that career exploration and decision self-efficacy have the significant mediating effect on the relationship between grit and career adaptability. Similarly, positive affect and goal commitment have significant mediating effects on the relationship between grit and career adaptability, with 95% confidence intervals of (0.002, 0.022) and (0.005, 0.044), respectively. The results of this model prove that there are differences in the mechanism of self-regulation in the influence of grit on career adaptability.

**Table 4:** Results of total effect, direct benefit, and indirect benefit.

The path	Effect	Se	Bias correcting confidence intervals (Boot95%CI)	
			LLCI	ULCI
Total effect: Grit → Career adaptability	0.417	0.046	0.327	0.507
Direct effect: Grit → Career adaptability	0.287	0.043	0.204	0.371
Indirect effect: Grit → Career adaptability	0.130	0.027	0.077	0.184
Grit → Career exploration and decision self-efficacy → Career adaptability	0.096	0.023	0.052	0.141
Grit → Positive affect → Career adaptability	0.011	0.005	0.002	0.022
Grit → Goal commitment → Career adaptability	0.023	0.010	0.005	0.044

LLCI and ULCI are the lowest and highest values of confidence interval, respectively.

## Discussion

Previous studies have pointed out the direct impact of grit on career adaptability (Han, 2018). We will discuss the mediating mechanism and visualize it. Based on the SRP, we examine the relationship between grit and career adaptability for Chinese college students as sample and examine potential mediating mechanisms, career exploration and self-efficacy decision, positive affect, and goal commitment.

### Theoretical Implication

First of all, we find that grit can positively predict the career adaptability of Chinese college students. Previous studies have been based on samples from other countries (Gregor et al., 2021; Kim and Kong, 2021). We are based in China, and grit is a quality that has become the most concerned education topic in Chinese society and has been emphasized in Chinese culture, such as volition (Zhao et al., 2018). Therefore, it is necessary to study Chinese students’ grit to career adaptability. This suggests that in the Chinese context we can also improve the career adaptability of college students by cultivating their grit. Moreover, it is likely that college students with enthusiasm, perseverance, and long-term goals are better able to cope with predictable tasks and roles, and are better able to self-regulate to unpredictable results brought about by job changes, thus bringing positive results. This study verifies that grit also has a positive impact on Chinese college students’ career adaptability and expands the theoretical framework and scope of application of the relationship between grit and career adaptability.

In addition, career exploration and decision self-efficacy, positive affect, and goal commitment have been proved to mediate the effect of grit on Chinese college students’ career adaptability. That is to say, Chinese college students with high grit can actively conduct career exploration, have stronger belief in their success in making career decisions, maintain positive affect and a high goal commitment to self-regulate, and thus improve their career adaptability. This study attempts to extend the application of SRP to the field of personal career development and has achieved some results, which be propitious to support the self-regulation theory and provide a reference for subsequent researches.

### Practical Implications

For college students, learning in college is crucial. Whether they can find a job they like and can be qualified for lies in their self-planning in college, as well as the cultivation of counselors, teachers, and parents. This study found grit to be significantly linked to career adaptability, and SRPs act as the important mediating mechanism between grit and adaptability. Therefore, the application of this research results involves the importance of developing persistence and consistency in students. In addition, in order to improve college students’ career adaptability, we should start from two aspects of cultivating grit and improving students’ SRPs.

When college students are looking for jobs, they may face difficulties in employment, do not want to find a job, and do not know their career goals and other problems. We should be targeted at individuals with high or low grit. Specifically, when confronted with career difficulties, students with high grit should improve their SRPs and understand their career preferences through practice. First, students should improve their sense of self-efficacy, set up quantifiable, time-bound and challenging career goals, and maintain a high level of effort and self-belief. Secondly, after setting appropriate career goals, you can timely adjust and improve your goals by analyzing problems. Do not give up easily and believe that you can achieve your goals. Finally, we should also manage our emotions and learn to control them. When the negative emotions accumulate to a certain extent, students should find some ways to vent the bad emotions, such as running or mountain climbing, and do something meaningful in their spare time to improve their SRP and activate the positive emotions effectively.

On the contrary, students with low grit need guidance from counselors and teachers, so as to help them improve grit and career adaptability. Teachers and counselors can implement educational and counseling interventions to cultivate college students’ grit. When students encounter setbacks in the process of career exploration and development, it is more necessary for counselors and teachers to encourage them to have the courage to continue on the road ahead. At the same time, counselors and teachers also need to teach students how to face all kinds of troubles and pressures in the career exploration and development. In particular, counselors and teachers can help students improve their SRPs. When students encounter difficulties. Frist, counselors and teachers need to help students recover their self-efficacy and participate in exploring their abilities and other career possibilities, so as to help them better adapt to career development and make better career choice. Secondly, counselors and teachers can help students set goals correctly. If the goal is considered achievable, then it is necessary to formulate specific strategies to achieve the goal. If the goal does not fit the student, help them set a more suitable goal to prevent students from repeating failure. Third, counselors and teachers should communicate effectively with students to ensure that students have the positive emotions during the process and remain positive toward their goals.

Parents can also encourage college students’ grit, as well as open experience to seek knowledge and explore education and career options, thus helping them make career decisions and better adapt to future career development. First, parents play an important role in influencing their children’s interests and ideas, supporting all efforts to achieve their goals and improve students’ decision self-efficacy in the form of praise. In addition, parents are the main source of vocational knowledge for children, providing information materials on jobs, attending various career development seminars, or providing information on aptitude and interest tests. Parents can help them set career goals that suit them and encourage them to stick with them. Finally, parents can support their children’s decisions through communication, express their support and interest in their children’s career, help their children find emotional outlets and the right way of expression, be a listener, stand in their children’s point of view to experience their feelings and ideas, and express their understanding of their children.

### Limitations and Future Directions

First, our measures of grit and career adaptability rely only on self-reported measures, so the scale that generated the scores in our study may be affected by general methodological biases. Future research needs to use the longitudinal design method of self-report, parental report, and outcome variable behavioral assessment to explore the influencing factors and the mediating role of grit on more career outcomes in the process of career development.

Secondly, it is also essential to study the boundary conditions of these relationships. Self-regulation theory proposes that environment (e.g., social support) and basic characteristics of goals (e.g., goal importance) shape people’s self-regulating behavior and influencs their career outcomes (Lent et al., 2017; Hu et al., 2018). For example, parents’ cultivation of grit and a harmonious family atmosphere make students more likely to adjust their behavior or goals to adapt their career development. These possibilities need to be validated in future studies.

Thirdly, the research sample has some limitations. The samples of this study are college students in southwest China, so whether the theoretical model proposed by this study is universally applicable to sample groups from other regions or with different educational backgrounds remains to be further verified. Future studies can expand the research sample, and compare whether there are differences in the influence mechanism of grit and SRP on career adaptability in samples from different regions, so as to promote the validity and consistency of research conclusions.

## Data Availability Statement

The original contributions presented in the study are included in the article/supplementary material, and further inquiries can be directed to the corresponding authors.

## Ethics Statement

Ethical review and approval was not required for the study on human participants in accordance with the local legislation and institutional requirements. The patients/participants provided their written informed consent to participate in this study in line with the Declaration of Helsinki. Research respondents were ensured confidentiality and anonymity. All participation was voluntary.

## Author Contributions

HL wrote the manuscript and analyzed the data under the guidance of XY and YM. XL contributed to data analysis and editing of the manuscript. XY and YM contributed to study design and data collection. LL and NL contributed to study design and critical revisions. All authors contributed to the article and approved the submitted version.

## Funding

This work was supported by Social Science Planning Cultivation Project of Chongqing (2020PY38), the Youth Project of National Natural Science Foundation of China (71802033), the Graduate Innovation Project of Chongqing (CYS21386), and the Key Research Base of Philosophy and Social Sciences in Sichuan Province-Social Governance Innovation Research Center Project (SHZLYB19009), Humanities and Social Sciences Research Project of Chongqing Municipal Education Commission Foundation (21SKGH125), Scientific Research Project of Chongqing Technology and Business University (2155028), and Open Funding of Chongqing Technology and Business university (KFJJ2019050).

## Conflict of Interest

The authors declare that the submitted work was carried out in the absence of any commercial or financial relationships that could be construed as a potential conflict of interest.

## Publisher’s Note

All claims expressed in this article are solely those of the authors and do not necessarily represent those of their affiliated organizations, or those of the publisher, the editors and the reviewers. Any product that may be evaluated in this article, or claim that may be made by its manufacturer, is not guaranteed or endorsed by the publisher.
